# LTVAE: Hybrid LSTM–Transformer VAE Architecture for Molecular SMILES Representation

**DOI:** 10.1002/jcc.70521

**Published:** 2026-11-03

**Authors:** Md Halim Mondol, Qingshan Wei, Edgar Lobaton

**Affiliations:** ^1^ Electrical and Computer Engineering North Carolina State University Raleigh North Carolina USA; ^2^ Chemical and Biomolecular Engineering North Carolina State University Raleigh North Carolina USA

**Keywords:** deep learning for chemistry, latent space analysis, molecular generative models, molecular representation learning, SMILES‐based modeling, variational autoencoders

## Abstract

A hybrid LSTM–Transformer Variational Autoencoder (LTVAE) framework is introduced in this paper for learning compact and chemically meaningful latent representations from SMILES strings, providing a SMILES‐to‐latent‐to‐SMILES representation model trained on ZINC15 and evaluated on ZINC15 and an out‐of‐distribution dye dataset. In the proposed model, sequential and structural dependencies in molecular strings are encoded using a bidirectional LSTM, while a Transformer‐based decoder is employed to reconstruct valid molecular structures in the evaluated settings. When trained on a curated ZINC15 dataset, the framework achieves reasonable reconstruction performance, with 98.58% token‐level accuracy, 92.19% string‐level accuracy, a 99.30% chemical validity ratio, and an average Levenshtein distance of 0.29 using a 64‐dimensional latent space. Reconstruction under an out‐of‐distribution evaluation setting is evaluated on a structurally distinct dye dataset, where 95.56% token‐level accuracy and 85.30% validity are achieved. The learned latent space preserves trends aligned with both general molecular descriptors and dye‐relevant structural descriptors, particularly conjugation length and aromatic surface fraction, with Fsp 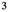 providing complementary information on molecular saturation. Controlled perturbations in the latent space suggest smooth and chemically consistent structural variations, illustrating the continuity and interpretability of the learned latent manifold.

## Graphical abstract

LTVAE integrates a bidirectional LSTM encoder with a Transformer decoder to learn compact molecular representations from SMILES. The latent space preserves key structural features and supports robust molecular reconstruction.

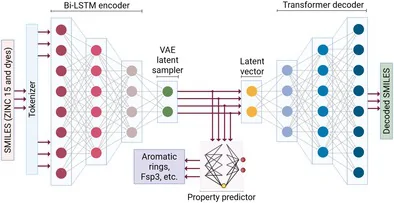

## 1 Introduction

Learning compact and chemically meaningful representations of molecules is a central challenge in computational chemistry and molecular machine learning [1]. Effective molecular representations enable tasks such as structure reconstruction, property analysis, similarity assessment, and controlled molecular generation, while reducing reliance on handcrafted descriptors or domain‐specific feature engineering [2]. In recent years, deep generative models, particularly variational autoencoders (VAEs), have emerged as powerful frameworks for learning continuous latent spaces that capture the underlying structure of molecular data [3]. When combined with string‐based molecular representations such as SMILES, these models offer a flexible and scalable approach to representation learning within the molecular distributions represented in training and evaluation datasets without requiring explicit graph construction or expert‐designed fingerprints [4].

To advance molecular representation learning beyond handcrafted descriptors, machine learning (ML) and deep learning (DL) methodologies have increasingly been adopted to model molecular structure directly from data [5]. Early approaches relied on fixed molecular fingerprints or physicochemical descriptors, which often fail to capture subtle structural variations or sequential dependencies [6]. More recently, sequence‐based molecular learning frameworks that operate directly on SMILES representations have gained prominence due to their ability to learn expressive representations without explicit feature engineering [7]. These approaches naturally align with neural sequence models and enable end‐to‐end learning of latent molecular representations within SMILES‐based molecular datasets.

Generative latent‐variable models [8], particularly VAEs [9, 10], and Generative Adversarial Networks (GANs) [11], have demonstrated strong performance in molecular representation learning and novel compound generation. Transformer‐based models trained on large‐scale SMILES datasets [12], such as SMILES Transformer [13, 14] and MoLFormer [15], as well as recent graph‐based variational architectures [16], have further advanced molecular property prediction and structure generation. Collectively, these works highlight a key requirement in molecule generation and downstream predictive tasks: accurately encoding SMILES into a continuous latent representation and reliably reconstructing them back. Failures in either direction introduce syntactic or structural artifacts that propagate errors into later prediction stages.

Figure 1 provides a conceptual overview of the proposed model. The encoder utilizes a lightweight bidirectional LSTM to extract sequential and structural dependencies from SMILES strings, while the Transformer‐based decoder leverages self‐attention to reconstruct molecular structures with high fidelity. This design allows the latent representation to remain compact while retaining chemically meaningful structure‐level information, making the model computationally efficient and scalable to large chemical libraries.

**FIGURE 1:**
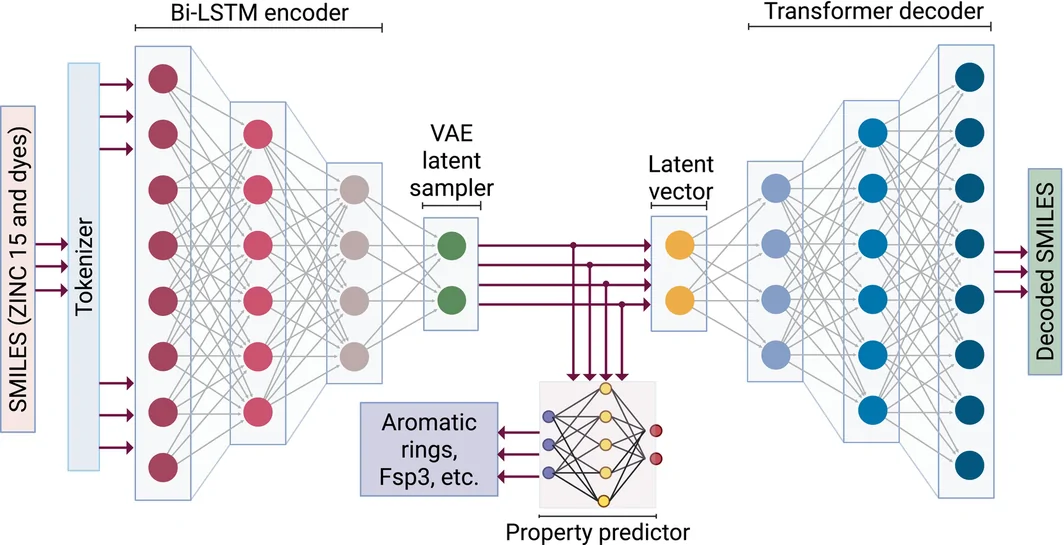
Overview of the proposed hybrid LSTM–Transformer VAE (LTVAE) model architecture. The BiLSTM encoder maps tokenized SMILES into latent vectors, which are subsequently decoded by a Transformer to reconstruct valid molecular structures.

Building upon this foundation, this work introduces a hybrid LTVAE framework designed for learning compact and chemically meaningful latent representations from SMILES strings. The model achieves a reconstruction accuracy of 98.58%, a chemical validity ratio of 99.30%, and an average Levenshtein distance of 0.29, demonstrating strong reconstruction performance on ZINC15 test molecules. The resulting latent space supports smooth exploration of molecular diversity and enables controlled generation of structurally related analogs, highlighting reconstruction robustness under the in‐distribution and out‐of‐distribution (OOD) evaluation settings used here. Because the OOD evaluation set is dye‐related, the latent‐space analysis includes dye‐relevant descriptors such as conjugation length (CL) and aromatic surface fraction (ASF) in addition to conventional molecular descriptors.

## 2 Related Work

Molecular representation learning has witnessed rapid growth through deep generative modeling, enabling efficient navigation of chemical space for design and discovery. Early approaches such as *Mol2Vec* [17] introduced unsupervised, chemistry‐aware embeddings by adapting the Word2Vec paradigm to molecular substructures. This technique converted atom‐centered fragments derived from Morgan fingerprints into dense, continuous vectors that capture underlying chemical relationships. Although Mol2Vec provided a scalable and interpretable feature space, it lacked explicit reconstruction capability, limiting its generative utility for de novo molecule design.

A major milestone was achieved by Gómez‐Bombarelli et al. [9], who introduced the first VAE framework for molecular design using SMILES representations. Their encoder–decoder architecture mapped discrete molecules into a continuous latent space, enabling gradient‐based optimization for inverse design. The model demonstrated the feasibility of continuous molecular interpolation and property‐driven search, laying the groundwork for subsequent developments in deep generative chemistry. However, reconstruction validity remained moderate, and the model struggled with long or syntactically complex SMILES strings due to the sequential nature of RNN‐based decoders.

Building on these foundations, hybrid and attention‐based architectures emerged to improve both expressivity and chemical validity. Transformer‐VAE, a Transformer‐based VAE proposed by Yoshikai et al. [18], reconciled the mismatch between VAE's probabilistic formulation and Transformer's sequence‐to‐sequence attention mechanism. Their model achieved superior generalization, generating previously unseen molecular scaffolds and enabling property prediction directly from latent variables. Notably, the authors reported that latent dimensionality could be reduced to as low as 32 without compromising reconstruction, emphasizing the potential for compact molecular descriptors. Similarly, TVAE employs a graph neural network encoder together with a Transformer decoder, as described by Bhadwal et al. [19], and is treated here as a separate baseline from Transformer‐VAE, demonstrating improved validity and novelty while addressing posterior collapse through architectural regularization. However, these architectures were primarily designed for broad molecular generation tasks and rely on Transformer or graph‐based encoders, introducing higher computational and parameter overhead that can be suboptimal for parameter‐efficient representation learning or chemical interaction applications. While prior Transformer‐based VAEs have explored latent dimensionality reduction, such analyses were conducted in conjunction with comparatively heavy encoder architectures. In contrast, the impact of aggressively compressing latent representations under a lightweight sequential encoder has not been systematically examined.

Parallel to these developments, domain‐specific implementations have leveraged VAE frameworks for property‐driven molecule design. Yalamanchi et al. [20] developed a β‐VAE for learning molecular representations of fuels, employing hierarchical priors and KL annealing to balance reconstruction fidelity and latent disentanglement. Their results underscored the importance of well‐structured latent spaces for property prediction tasks such as octane rating and emission potential. Despite these advances, most existing models were trained on drug‐like or hydrocarbon datasets, leaving structurally compact and chemically distinct molecular classes—such as small dyes—relatively underexplored in terms of OOD reconstruction robustness.

Collectively, these studies have demonstrated that generative learning can effectively capture chemical structure and property correlations through latent representations. However, challenges persist in achieving high reconstruction fidelity and validity while maintaining model compactness and robustness beyond a given training distribution. In contrast to existing Transformer‐based VAEs, which have been developed predominantly for large, drug‐like molecular corpora, the proposed framework is designed as a ZINC15‐trained SMILES‐to‐latent‐to‐SMILES representation model with an emphasis on parameter efficiency and reconstruction robustness under OOD evaluation. A key architectural distinction lies in the encoder design: rather than employing a full Transformer or graph‐based encoder, a lightweight BiLSTM encoder is used in conjunction with a variational bottleneck and a Transformer decoder. This design explicitly targets efficient sequential SMILES encoding while avoiding the computational and parameter overhead associated with heavy attention‐based or graph‐centric encoders. Despite its reduced parameter count, the proposed encoder captures essential sequential and structural dependencies and achieves reconstruction accuracy, chemical validity, and structural similarity comparable to, and in several cases exceeding, those reported for heavier Transformer‐based VAEs. Evaluation on a structurally distinct dye dataset is included as an OOD test case to assess reconstruction robustness, demonstrating that the learned latent representation remains stable and expressive beyond the ZINC15 training distribution. This balance between parameter efficiency, reconstruction fidelity, and reconstruction robustness under an OOD evaluation setting fills an important gap in existing hybrid generative models.

## 3 Methodology

### 3.1 Problem Statement

The broader motivation of this research is to enable reconstruction‐focused SMILES representation learning from ZINC15‐trained models that can support the downstream evaluation tasks considered here. A critical prerequisite for such capabilities is a molecular representation model that can reliably encode and decode SMILES strings while preserving chemically meaningful information within a continuous latent space. The work presented in this paper is focused specifically on establishing this foundational capability. To this end, a hybrid BiLSTM–VAE encoder coupled with a Transformer decoder is designed, trained, and evaluated to achieve high‐fidelity SMILES‐to‐latent‐to‐SMILES mappings. By prioritizing reconstruction accuracy, validity, latent compactness, and interpretability, this study establishes a SMILES latent representation framework trained on ZINC15 and evaluated for reconstruction robustness beyond the training distribution. The following section details the datasets and architectural choices used to realize this objective.

### 3.2 Dataset

The chemical dataset utilized for model training and validation in this study was sourced from the ZINC15 database [21], which provides a large collection of commercially available molecules. All SMILES strings were first filtered to remove invalid entries and then canonicalized to ensure uniqueness and eliminate redundancy. The maximum SMILES length observed after preprocessing was 149 characters, which informed the sequence length used during model training.

From the cleaned corpus, 250 k SMILES were randomly sampled for training and 80 k SMILES were randomly sampled for validation. Random sampling was intentionally adopted to sample representative molecules within the curated ZINC15 corpus while avoiding bias toward any specific molecular class or scaffold. This choice was important to encourage learning of ZINC15‐based SMILES representations that could be evaluated for reconstruction robustness on a structurally distinct OOD dye dataset. Prior to training, 270 SMILES were removed from the validation pool and reserved as a held‐out primary test set, ensuring strict separation between training, validation, and test data.

In addition to the ZINC15‐derived splits, a second curated test set was constructed from a collection of dye molecules containing 34 chemically distinct SMILES. Molecular dyes are a class of functional compounds widely used in optical, electronic, and chemical sensing applications, including colorimetric and fluorescent detection of volatile organic compounds (VOCs) [22]. Their sensing functionality typically arises from extended π‐conjugation, rigid aromatic frameworks, and the presence of heteroatoms or charged functional groups that enable strong interactions with analyte molecules [23]. In this work, the dye dataset is employed exclusively as an application‐motivated OOD test set to evaluate reconstruction robustness beyond the ZINC15 training distribution for molecules relevant to VOC sensor design.

Although both the ZINC15 molecules and the dye dataset are represented using SMILES strings, they correspond to fundamentally different chemical distributions. ZINC15 predominantly consists of small‐ to medium‐sized, drug‐like molecules characterized by flexible backbones and a balanced composition of common organic elements such as C, O, N, S, and halogens. In contrast, dye molecules are typically larger, more rigid, and highly conjugated, often containing extended aromatic systems, heterocycles, formal charges, or metal‐associated motifs. These structural characteristics result in longer SMILES sequences, distinct token frequency distributions, and chemical features that are sparsely represented in drug‐like molecular libraries. Consequently, dye SMILES naturally occupy regions of chemical space that differ from the dominant ZINC15 manifold, making them a chemically distinct OOD evaluation set for reconstruction robustness.

Dyes with excessively long SMILES strings exceeding the maximum sequence length were excluded to ensure compatibility with the trained model. Prior to training, all SMILES were tokenized into a vocabulary of 69 distinct tokens, preserving multi‐character atomic symbols and stereochemical indicators (e.g., “@@”, “Na”, “Cl”) as single tokens to maintain chemically faithful sequence representations.

To further examine how the learned latent space organizes structurally distinct molecules relative to the ZINC15‐derived corpus, a latent‐space proximity analysis was performed. Rather than projecting the full dataset, molecules were filtered from the combined pool based on their proximity to a selected dye test subset in latent space, forming a focused neighborhood. This subset captures molecules that are locally relevant to the dye chemical manifold while remaining diverse in structure. Subsequent PCA and property analyses were conducted on this filtered set to investigate latent–property correlations and local continuity within these neighborhoods. This strategy enables targeted interpretation of the latent manifold in chemically meaningful regions without assuming global overlap with the full ZINC15 distribution.

### 3.3 Model Architecture

The detailed internal diagram of the proposed LTVAE architecture, shown in Figure 2, integrates the sequential modeling strengths of recurrent networks with the global contextual awareness of Transformer decoders. The model efficiently encodes SMILES strings into continuous latent vectors and reconstructs them with high structural fidelity.

**FIGURE 2:**
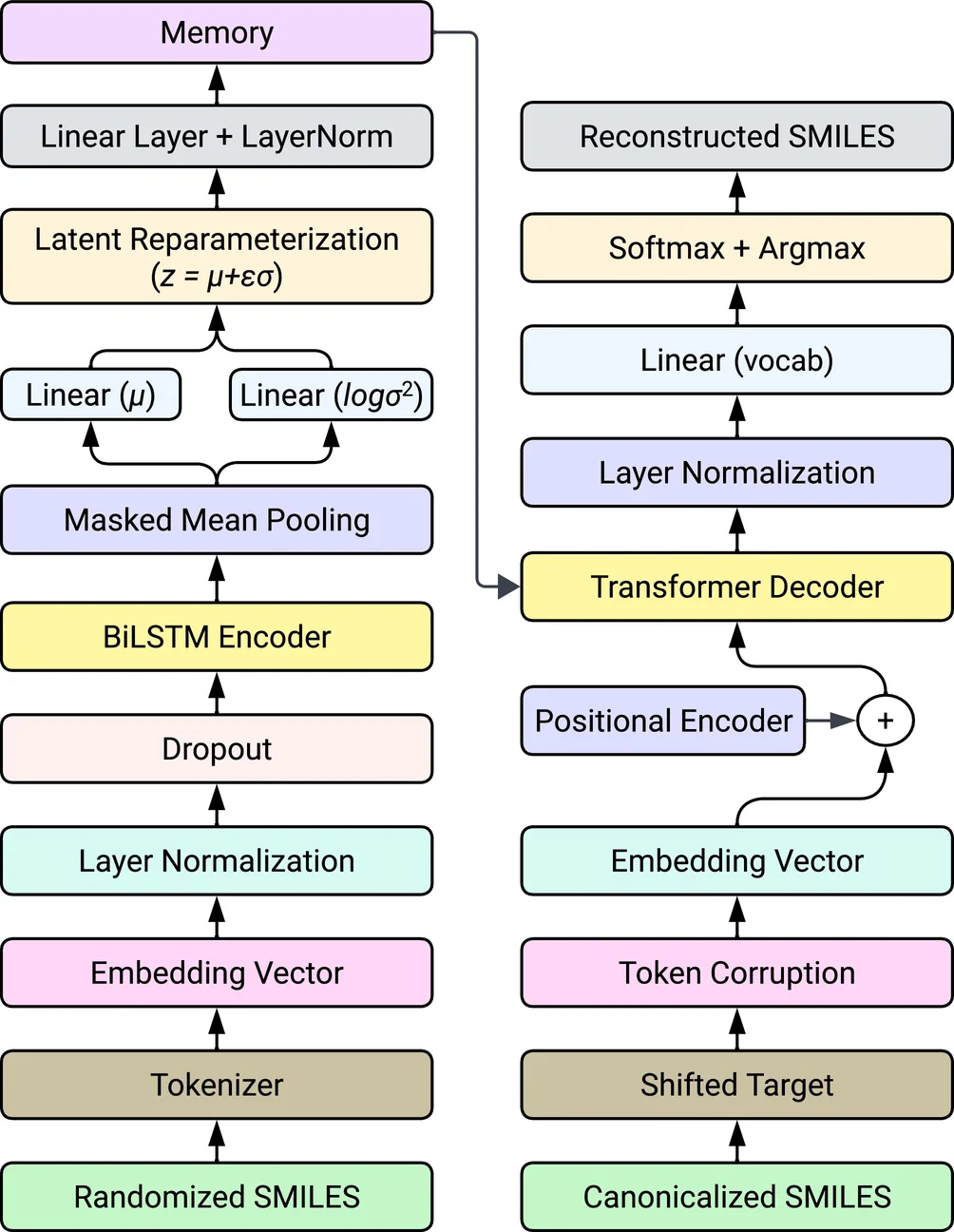
Internal block diagram of the hybrid LTVAE model.

During the encoding phase, the input SMILES, converted into randomized SMILES through atom renumbering, are tokenized and embedded into continuous vector representations. These embeddings are subsequently regularized using *Layer Normalization* and standard layer‐wise *dropout* to mitigate overfitting and stabilize training. The inclusion of randomized SMILES improves encoding robustness by reducing sensitivity to SMILES ordering, leading to more stable reconstruction behavior compared to using canonical SMILES alone. During training, one randomized SMILES representation per molecule is sampled on‐the‐fly at each epoch and used exclusively as encoder input, while the corresponding canonical SMILES are retained as decoder targets. This SMILES augmentation strategy promotes invariance of the learned latent representation to SMILES ordering without artificially increasing the effective dataset size.

These embeddings are processed by a lightweight BiLSTM encoder that captures long‐range dependencies in both directions of the molecular sequence. The resulting sequence features are aggregated into a single fixed‐length vector using masked mean pooling, ensuring uniform latent dimensionality independent of input length. Two parallel fully connected layers generate the latent distribution parameters—the mean (μ) and log‐variance ($\log\sigma^{2}$)—which define the variational space. Through the reparameterization trick (z=μ+ϵσ), a differentiable latent sample is drawn and projected to the Transformer model dimension via a linear layer with *Layer Normalization*. This projected vector serves as the single‐token memory input to the decoder.

On the decoding side, canonicalized SMILES strings are used as target sequences during training. The decoder employs teacher forcing, where ground‐truth tokens are provided as inputs at each time step, shifted right by one position. To enhance robustness, token‐level corruption is introduced with a small probability, encouraging the model to recover from noisy or incomplete inputs. The decoder first embeds the input sequence and applies sinusoidal positional encoding before feeding the representations into a multi‐layer Transformer decoder with causal masking. Finally, the output passes through *Layer Normalization* and a linear projection to the vocabulary space, followed by a *softmax* activation to produce probability distributions over SMILES tokens. Inference is performed using beam search decoding to explore multiple reconstruction paths and maximize sequence validity and fidelity.

The design choice of a BiLSTM encoder coupled with a Transformer decoder was motivated by the need to balance parameter efficiency with expressive generative capability. While prior work has explored combinations of recurrent and attention‐based models, the specific integration of an LSTM‐based sequential encoder with a Transformer decoder through a variational bottleneck has not been systematically examined for SMILES‐based molecular representation learning. The proposed architecture enables efficient encoding of sequential SMILES structure using a compact recurrent encoder, while retaining the global reconstruction capacity of a Transformer‐based decoder. As demonstrated in the comparative analysis, high reconstruction fidelity and chemical validity are achieved despite a substantially reduced encoder parameter count, addressing an important architectural gap in existing Transformer‐based VAE hybrids and supporting scalable SMILES representation learning within the evaluated ZINC15‐trained and OOD dye reconstruction setting.

### 3.4 Training and Validation

During training and standard validation, teacher forcing was applied throughout all epochs, feeding the correct ground‐truth token at each decoding step to guide the sequence generation process. Every five epochs, the model was evaluated under non‐forced conditions using beam search decoding with a beam width of 5. This periodic inference enabled assessment of reconstruction accuracy, validity ratio, and Levenshtein distance in realistic generative settings.

The model was optimized using a composite loss function combining token‐level categorical cross‐entropy with a weighted Kullback–Leibler (KL) divergence term, following the standard VAE objective. Specifically, the training loss is defined as, [24]

(1) $$\begin{array}{c} L=E_{q_{\phi }\left(z|x\right)}\left[L_{\mathrm{rec}}\left(x,\hat{x}\right)\right]+\beta \;D_{\mathrm{KL}}\;\left(q_{\phi }\left(z|x\right)∥p\left(z\right)\right), \end{array}$$

where $L_{\mathrm{rec}}$ denotes the token‐level categorical cross‐entropy reconstruction loss between the input SMILES x and the reconstructed sequence $\hat{x}$, $q_{\phi }\left(z|x\right)$ is the approximate posterior, and $p\left(z\right)$ is the standard normal prior over the latent variables. A gradual KL annealing schedule increased the weighting coefficient (β) up to 0.02, stabilizing the trade‐off between reconstruction fidelity and latent regularization.

Label smoothing was applied to mitigate overconfidence in token predictions, while decoder input corruption was applied at the token level by randomly replacing non‐special input tokens, encouraging the decoder to recover from incomplete sequences. This mechanism is distinct from standard dropout, which was applied within network layers to regularize hidden representations. In addition, the use of randomized SMILES at the encoder was observed to improve reconstruction robustness and reduce overfitting, particularly for smaller latent dimensions, by encouraging the model to learn structure‐aware embeddings rather than sequence‐specific patterns. To assess the combined contribution of these augmentation and regularization strategies, Figure S3 in the Additional Information compares reconstruction performance on validation, ZINC15‐like test, and dye test sets with SMILES randomization, decoder token corruption, and label smoothing jointly enabled or disabled. Alongside these strategies, early stopping and an adaptive learning rate scheduler were used to promote convergence stability and limit overfitting.

The principal training and architectural hyperparameters used for model optimization are summarized in Table 1, and the source code of the trained model can be accessed from our repository. These settings were carefully chosen to balance computational efficiency and reconstruction accuracy, ensuring stable convergence while maintaining a smooth and densely populated latent space suitable for downstream generative and predictive tasks.

**TABLE 1:** Key hyperparameters and training configurations of the LTVAE model.

Parameter	Value/Description
Embedding dimension ($d_{\text{model}}$)	256
Latent dimension (z)	64
Encoder layers (BiLSTM)	7 layers, bidirectional
Decoder layers (Transformer)	7 layers, 8 attention heads
Dropout rate	0.05 (embeddings, LSTM, Transformer)
Decoder input token corruption ($p_{\text{corrupt}}$)	0.05
KL annealing maximum weight ($\beta_{\max}$)	0.02
Beam width (inference)	5
Optimizer	AdamW
Learning‐rate scheduler	Reduce‐on‐plateau
Early stopping patience	10 epochs
Label smoothing	Enabled (0.02)

### 3.5 Evaluation Metrics

The performance of the proposed model is assessed using four primary metrics: reconstruction accuracy, validity ratio, average Levenshtein distance, and property prediction performance. Reconstruction accuracy refers to the percentage of tokens in the output SMILES that are correctly predicted relative to the reference sequence, rather than requiring an exact match of the full sequence. The validity ratio measures the fraction of generated SMILES that correspond to chemically valid molecules, as verified by RDKit [25]. The average Levenshtein distance captures the mean token‐level edit distance between generated and reference SMILES, providing a fine‐grained measure of structural reconstruction quality. Property prediction performance is reported in terms of $R^{2}$ and mean absolute error (MAE) for both general molecular descriptors and dye‐relevant descriptors. The general descriptors include molecular weight (MW), aromatic rings (AR), Fsp 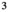, heavy atom count (HA), and rotatable bonds (RB), while the dye‐relevant descriptors include CL and ASF. This evaluation directly compares how well conventional structural features and dye‐aligned conjugation/aromaticity features are encoded in the learned latent vectors.

In addition to absolute performance, the effect of latent dimensionality on these metrics is examined to assess the compactness and expressiveness of the learned representation. The proposed architecture maintains high reconstruction accuracy and chemical validity even at reduced latent dimensions, in contrast to standard VAEs that often require larger latent spaces to achieve comparable performance. Furthermore, the LTVAE is compared against baseline generative models, including a standard SMILES‐based VAE, TVAE, and Transformer‐VAE, on both the ZINC15 dataset and a structurally distinct dye dataset used as an OOD test case to evaluate reconstruction robustness beyond the ZINC15 training distribution.

## 4 Results

### 4.1 Performance Evaluation

The reconstruction performance of the proposed LTVAE model was systematically evaluated across a range of latent space dimensionalities to assess both representational capacity and robustness. As shown in Figure 3, reconstruction accuracy, chemical validity, and average Levenshtein distance consistently improve as the latent dimension increases. A clear transition is observed between latent dimensions 16 and 32, beyond which the model rapidly achieves high reconstruction fidelity and stable validity, indicating that sufficient molecular information is captured in relatively compact latent spaces.

**FIGURE 3:**
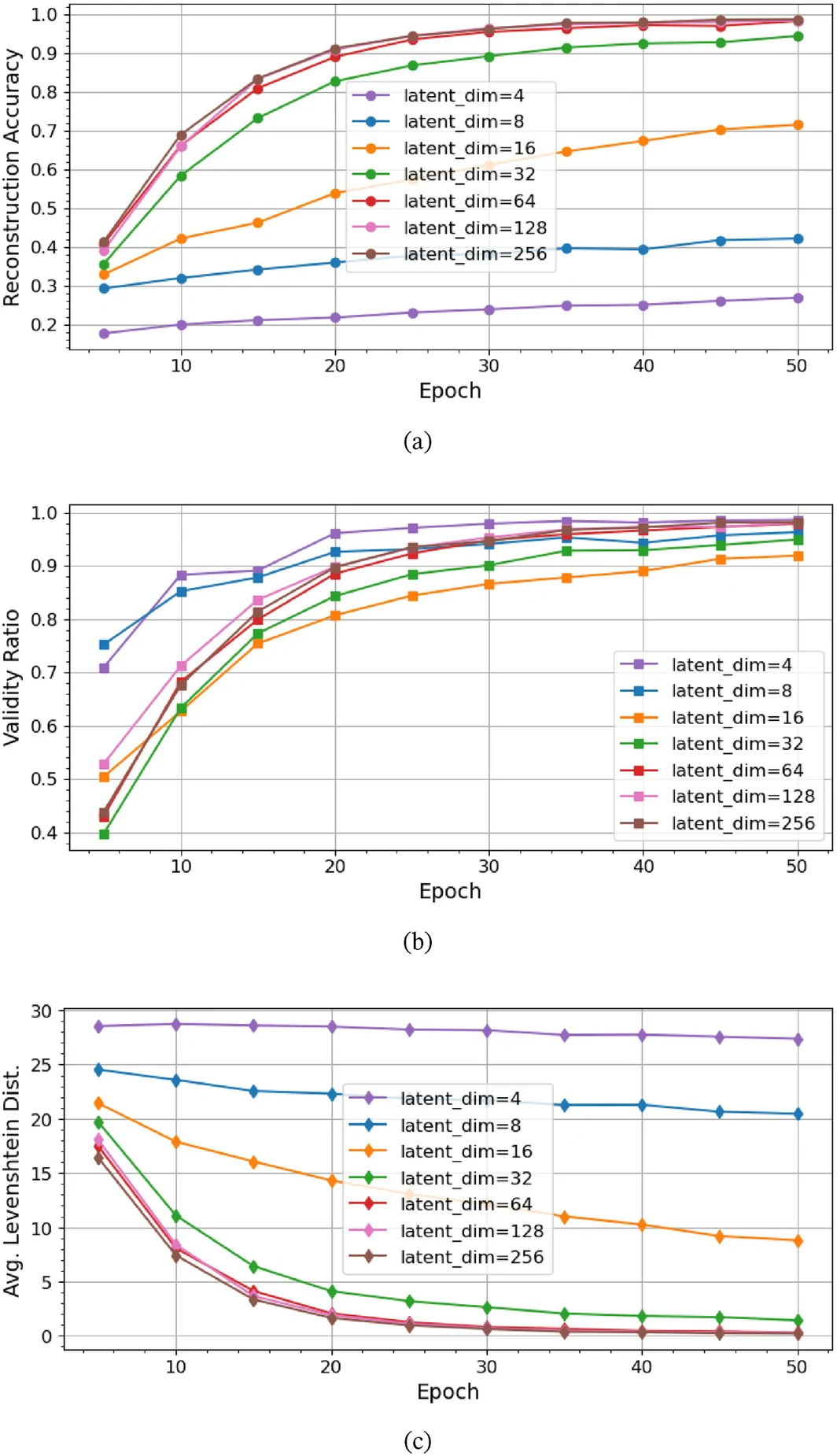
Performance of the proposed model on validation dataset: (a) validation accuracy, (b) chemical validity ratio, and (c) average Levenshtein distance.

Notably, strong reconstruction performance is maintained for latent dimensions as small as 32, with only marginal gains observed when increasing the latent size beyond 64. For latent dimensions ≥ 64, reconstruction accuracy exceeds 90% early in training, validity stabilizes near saturation, and Levenshtein distance approaches near‐zero values. These trends indicate diminishing returns from further latent expansion and highlight the efficiency of the proposed architecture. Unlike prior Transformer‐based VAEs that often rely on larger latent dimensionalities to preserve reconstruction quality, the proposed LTVAE achieves comparable fidelity with a compact latent representation, underscoring the effectiveness of the lightweight BiLSTM encoder in capturing essential SMILES structure.

Furthermore, the incorporation of KL annealing over the first 15 training epochs contributed to stable latent space organization. By gradually increasing the KL‐weighting coefficient, the model avoids premature collapse of the latent distribution while encouraging a well‐populated and continuous latent manifold. This structured latent space supports smooth interpolation and controlled molecular variation, which is critical for reliable latent‐space exploration and downstream generative or predictive tasks.

To evaluate in‐distribution reconstruction and reconstruction robustness under an out‐of‐distribution evaluation setting, the trained model was tested on two distinct datasets. On the ZINC 15 test set, the model achieved a token‐level reconstruction accuracy of 98.58%, a string‐level reconstruction accuracy of 92.19%, a validity ratio of 99.30%, and an average Levenshtein distance of 0.29, indicating that most reconstructed molecules were both syntactically valid and structurally consistent with their originals. When evaluated on an OOD test set containing more structurally specialized and less common molecular scaffolds, the model achieved a token‐level accuracy of 95.56%, string‐level accuracy of 61.76%, validity of 85.30%, and an average Levenshtein distance of 1.97. While performance is naturally lower on this more complex subset, the results reflect reconstruction robustness on a structurally distinct OOD dataset.

To investigate the variation in dye reconstruction performance, Figure S4 in the Additional Information examines reconstruction error in relation to nearest‐neighbor fingerprint‐ and SMILES‐based distances to a randomly sampled 100,000‐molecule ZINC15 reference set, rather than the original training subset. Complementing this assessment of proximity to the reference chemical space, Figure S5 in the Additional Information examines individual structural descriptors and shows weak linear associations of dye reconstruction error with CL, ASF, and SMILES length. To extend the evaluation beyond the ZINC15 and dye test sets, Table S3 in the Additional Information reports reconstruction performance for approximately 20,000 molecules across five PubChem domains not used for training: drug‐like compounds, pigment indicators, natural products, polymer monomers, and protein–enzyme chemistry molecules.

Beyond these reconstruction evaluations, the dye dataset was used as an application‐motivated case study to examine whether controlled latent‐space perturbations could generate chemically and practically plausible molecular analogs. Figure 4 presents representative input–output pairs obtained via controlled perturbations of latent vectors corresponding to dye molecules. Alongside each structure, MW, ASF, CL, Fsp 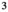, and synthetic accessibility SA scores (SAS) are reported, enabling comparison of both general molecular descriptors and dye‐relevant structural descriptors between inputs and generated analogs. The results show that core aromatic scaffolds and functional motifs are preserved, while modest and chemically reasonable variations are introduced. Notably, SA scores remain within a practical range and, in some cases, improve relative to the inputs, indicating that the generated molecules remain synthetically plausible. The preservation of aromaticity‐ and conjugation‐related descriptors indicates that latent perturbations retain structural features relevant to dye chemistry rather than only generic molecular property trends. Collectively, these analyses demonstrate that the learned latent manifold is smooth and chemically coherent, supporting controlled molecular exploration within this structurally distinct OOD dye dataset.

**FIGURE 4:**
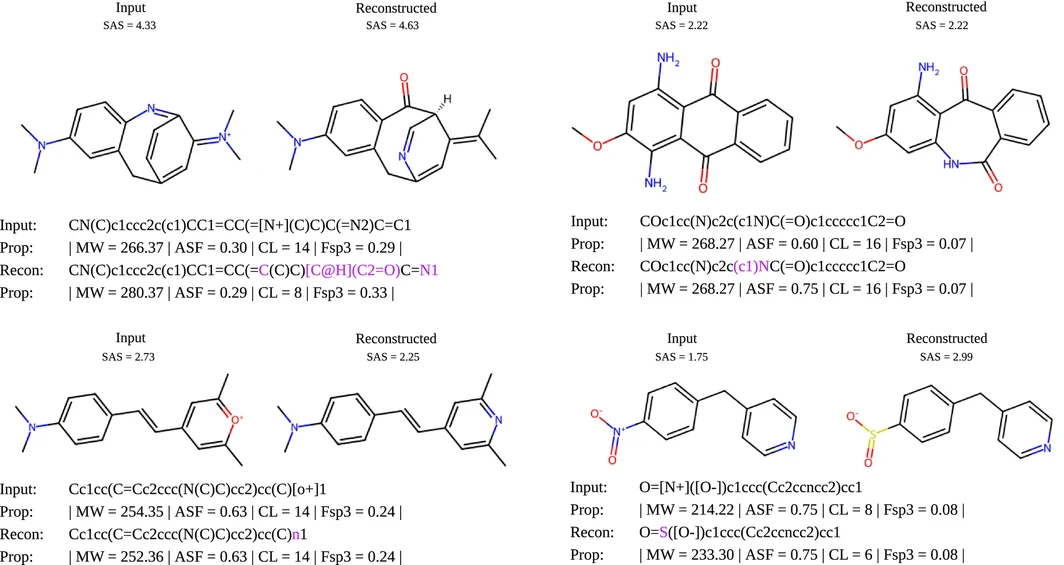
Examples of novel dye analogs generated through controlled sampling in the latent space of the proposed LTVAE model. Each pair shows an input dye (left) and a corresponding latent‐space‐perturbed counterpart (right), with deviations highlighted in the SMILES strings. For each molecule, molecular weight (MW), aromatic surface fraction (ASF), conjugation length (CL), Fsp 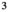, and synthetic accessibility score (SAS) are reported. The generated structures preserve core aromatic frameworks and conjugated motifs while exhibiting synthetically plausible modifications, as reflected by comparable SA scores and realistic shifts in dye‐relevant structural descriptors.

### 4.2 Performance on Property Prediction

Beyond reconstruction accuracy, the learned latent space was evaluated to determine whether chemically meaningful structural information is preserved. To this end, an additional subset of molecules was selected from the 20 M‐compound ZINC15 database by filtering molecules that lie in close proximity to dye and VOC embeddings in latent space. This subset represents a chemically relevant local neighborhood of the learned latent manifold. Property prediction was subsequently performed on the corresponding latent vectors to assess whether key molecular properties can be inferred directly from the learned representation. This analysis provides insight into the interpretability and practical utility of the latent space for molecular representation learning on structurally distinct test sets.

#### 4.2.1 Latent Space Visualization

Figure 5 presents the latent space projections obtained via principal component analysis (PCA), colored by CL, ASF, and Fsp 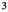. CL and ASF were selected because they reflect dye‐relevant conjugation and aromatic surface character, while Fsp 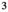 provides a complementary measure of molecular saturation and structural character. The smooth property gradients observed across the projected latent space indicate that the model organizes molecules along chemically meaningful directions connected to dye structure as well as general molecular geometry.

**FIGURE 5:**
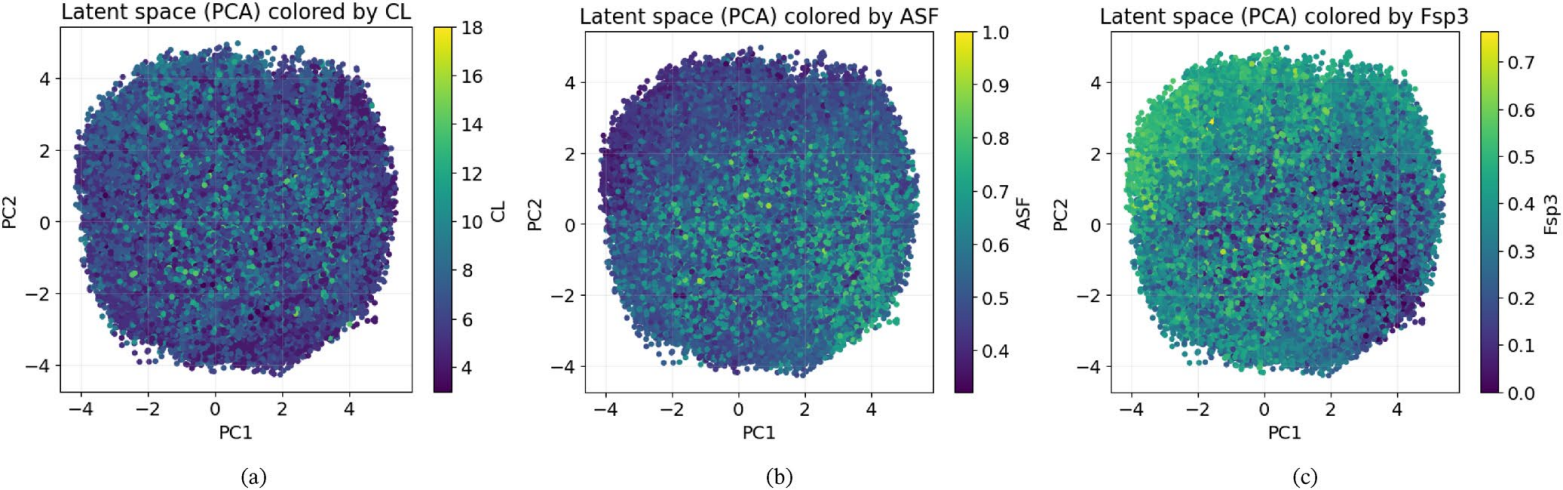
Latent space projections of the test molecules using PCA, colored by (a) conjugation length (CL), (b) aromatic surface fraction (ASF), and (c) Fsp 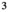. These descriptors capture dye‐relevant conjugation/aromaticity and complementary molecular saturation, showing that the learned latent space organizes chemically meaningful structural features beyond conventional drug‐discovery descriptors.

#### 4.2.2 Quantitative Property Prediction

To further quantify the chemical awareness of the latent space, ridge and multilayer perceptron (MLP) regressors were trained post hoc to predict selected molecular descriptors directly from the learned latent vectors. Table 2 compares general molecular descriptors with dye‐relevant descriptors, including MW, AR, ASF, Fsp 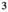, HA, RB, and CL. For MW, Ridge regression gives MAE = 12.585 and $R^{2}=0.480$, while the MLP improves performance to MAE = 5.230 and $R^{2}=0.900$. AR is predicted with Ridge MAE = 0.154 and $R^{2}=0.817$, and with MLP MAE = 0.005 and $R^{2}=0.997$. The dye‐relevant ASF descriptor is especially well captured, with Ridge MAE = 0.024 and $R^{2}=0.920$, and MLP MAE = 0.008 and $R^{2}=0.990$, indicating that the latent representation encodes aromaticity‐related dye structure. Fsp 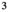 is also strongly predicted, with Ridge MAE = 0.033 and $R^{2}=0.897$, and MLP MAE = 0.014 and $R^{2}=0.980$. HA and RB show strong MLP performance as well, with MLP MAE/$R^{2}$ values of 0.201/0.978 and 0.304/0.924, respectively, compared with Ridge values of 0.479/0.881 and 0.717/0.623. For CL, Ridge performance is weaker (MAE = 1.420, $R^{2}=0.420$), whereas the nonlinear MLP reaches MAE = 0.487 and $R^{2}=0.918$, suggesting that conjugation information is present in the latent space but is encoded nonlinearly. Taken together, these results indicate that the MLP predicts the selected general and dye‐relevant descriptors well. To broaden this assessment of the information encoded in the latent vectors, Table S1 in the Additional Information extends the comparison to hydrogen‐bond acceptors (HBA), hydrogen‐bond donors (HBD), topological polar surface area (TPSA), log *P*, and QED.

**TABLE 2:** Prediction of molecular properties from latent vectors.

Property	Ridge		MLP	
	MAE	$R^{2}$	MAE	$R^{2}$
Molecular weight^a^	12.585	0.480	5.230	0.900
Aromatic rings	0.154	0.817	0.005	0.997
Aromatic surface fraction	0.024	0.920	0.008	0.990
Fsp 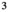	0.033	0.897	0.014	0.980
Heavy atom count (HA)	0.479	0.881	0.201	0.978
Rot bonds	0.717	0.623	0.304	0.924
Conjugation length	1.420	0.420	0.487	0.918

^a^ MAE for MW is reported in daltons (Da); all others are unitless.

#### 4.2.3 Latent‐Property Correlation

Pearson correlations were computed between PC1 and PC6 of the latent vectors and selected molecular properties, as shown in Figure 6. This analysis tests whether leading latent principal directions align with interpretable molecular descriptors. The heatmap includes both general descriptors and dye‐relevant descriptors, allowing the PCA‐based organization of size, saturation, aromaticity, ASF, and CL trends to be inspected without treating any single principal component as a complete property predictor. Moderate correlations support the presence of interpretable latent organization, while weaker entries are not over‐interpreted. Thus, Figure 6 complements, but does not replace, the quantitative property prediction results reported in Table 2. To assess whether a linear projection provides a meaningful visualization of the learned latent space, the variance explained by PCA was quantified. The first principal component explains approximately 7.00% of the total latent variance, while the first two components together account for approximately 12.30%. The first 10 principal components capture approximately 41.80% of the cumulative variance, as illustrated in Figure 7. These results indicate that the learned latent representations are distributed across multiple dimensions rather than concentrated along a small number of dominant axes, reflecting the high‐dimensional nature of the molecular embedding space. Consequently, the two‐dimensional PCA visualizations provide an informative qualitative projection for examining latent organization and property correlations, but they do not fully represent the complete latent structure. Importantly, all latent traversal experiments were performed directly in the original 64‐dimensional latent space rather than along PCA directions, ensuring that the observed property variations arise from the learned latent representations rather than artifacts of linear dimensionality reduction.

**FIGURE 6:**
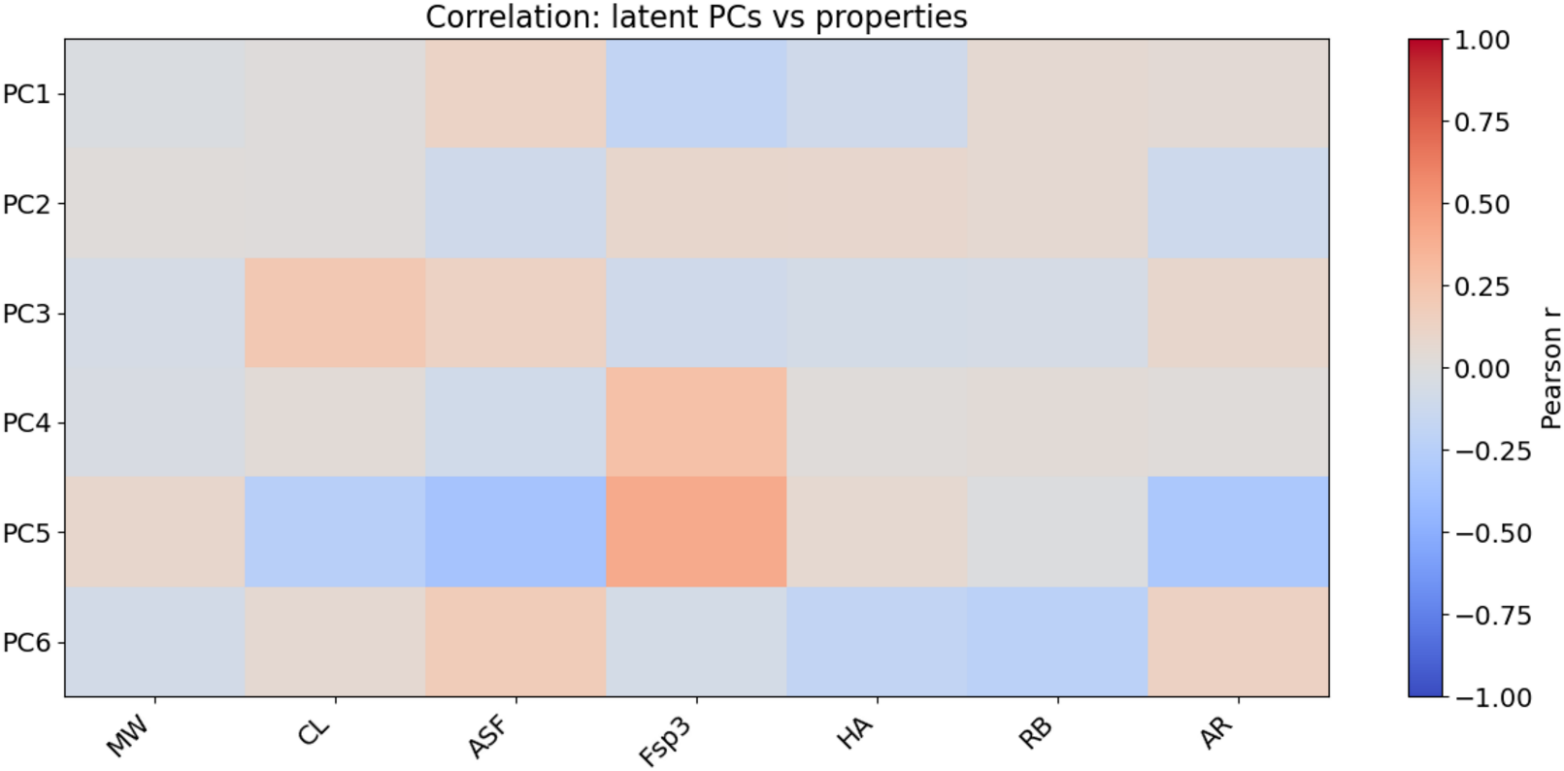
Correlation matrix showing Pearson r values between latent principal components PC1–PC6 and molecular properties. The properties include both general molecular descriptors and dye‐relevant descriptors, enabling comparison of how latent principal directions align with interpretable structural features.

**FIGURE 7:**
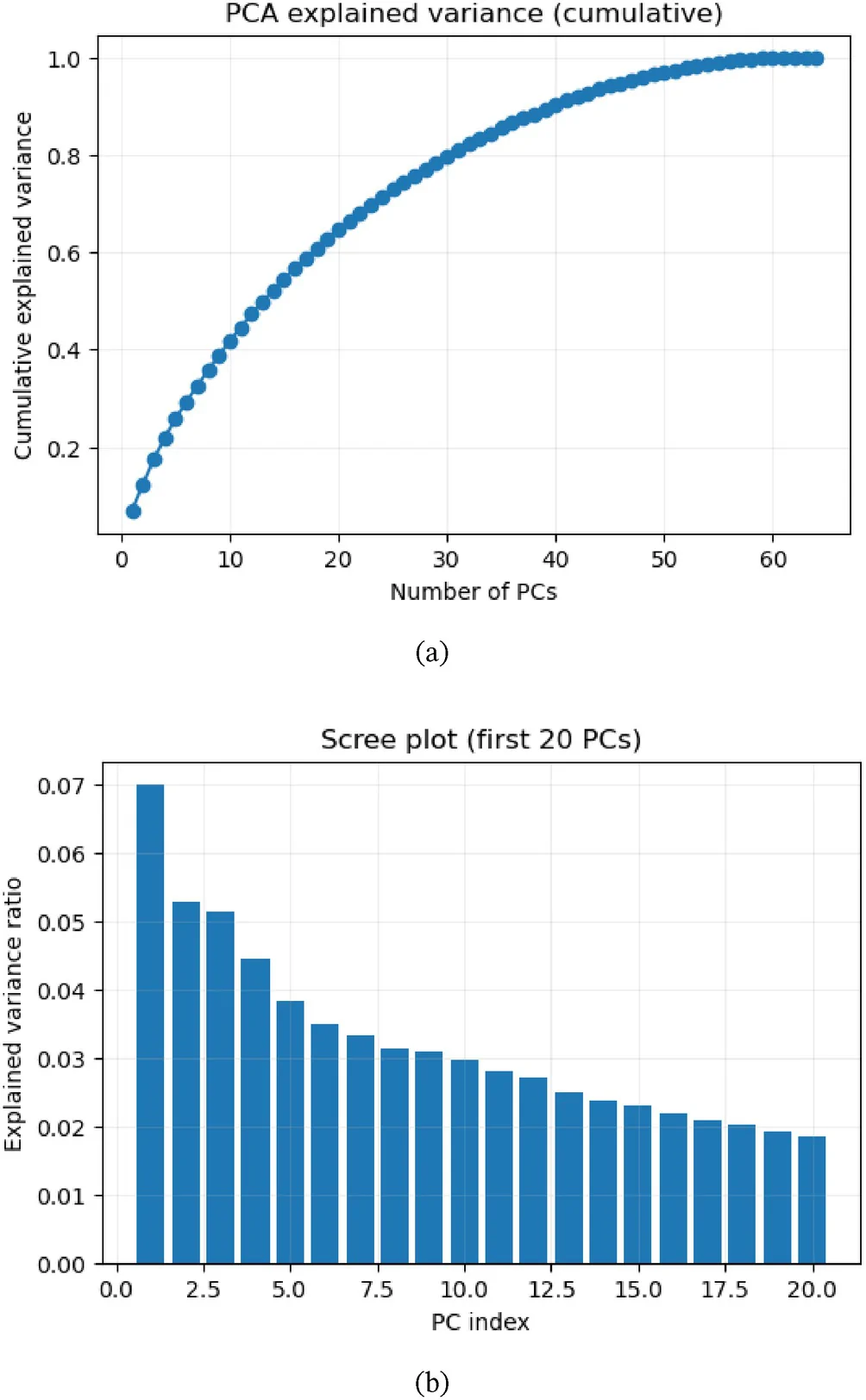
Explained variance analysis of the learned latent space using principal component analysis (PCA). (a) Cumulative explained variance as a function of the number of principal components. (b) Scree plot showing the variance contribution of the first 20 principal components. The explained variance is distributed across multiple principal components rather than concentrated in only the first few, reflecting the high‐dimensional nature of the learned latent representations. Consequently, PCA is used as a visualization tool to examine latent organization and property correlations, while latent traversal experiments are performed directly in the original latent space.

To further validate that these correlations correspond to continuous and controllable variations in chemical structure, latent‐space traversal experiments were conducted along a learned CL direction. Starting from an anchor molecule, latent vectors were incrementally perturbed along this direction using a scalar traversal parameter t, such that the latent code was modified along the learned CL direction before decoding. As shown in Figure 8, the decoded structures remain chemically valid while CL increases from approximately 7 to 14 across the displayed examples. This behavior supports property‐conditioned traversal for a dye‐relevant descriptor and indicates that the learned latent manifold encodes conjugation‐related structural variation in a locally continuous manner. Extending this example to other property directions, Figure S1 in the Additional Information presents the complete CL traversal alongside ASF and MW traversals, with decoded structures and their measured property values.

**FIGURE 8:**
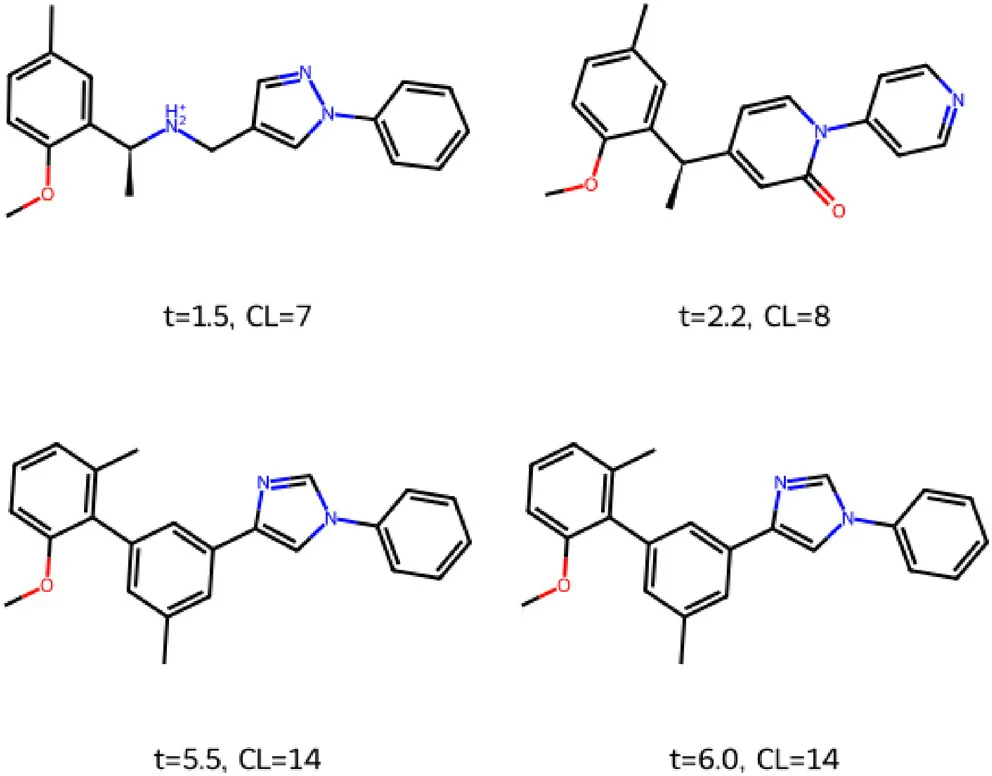
Latent‐space traversal along a learned CL direction. Starting from an anchor molecule, latent vectors were incrementally perturbed using a traversal parameter t and decoded to generate chemically valid structures. The resulting molecules exhibit smooth changes in conjugated structure, with CL increasing from approximately 7 to 14 in the displayed examples, illustrating dye‐relevant property‐conditioned organization of the learned latent manifold.

Overall, the combined correlation, variance, and latent traversal analyses confirm that the proposed LTVAE framework learns a structured and chemically meaningful latent representation. This latent manifold supports smooth, property‐aware molecular transformations while maintaining chemical validity, supporting molecular exploration within the evaluated ZINC15‐trained representation setting.

### 4.3 Comparative Analysis

The comparative evaluation in Table 3 assesses the proposed LTVAE framework relative to classical and Transformer‐based VAE architectures under both in‐distribution and out‐of‐distribution (OOD) conditions. In‐distribution performance is evaluated on ZINC15 test molecules drawn from the same ZINC15‐derived distribution as the training data, while reconstruction robustness under an OOD evaluation setting is examined using a structurally distinct dye dataset. In addition to a baseline SMILES‐based VAE, the comparison includes TVAE and Transformer‐VAE as representative Transformer‐based VAE variants with differing encoder designs, providing representative sequence‐ and attention‐based baselines. All models were implemented and trained under identical experimental conditions, including dataset splits, optimization schedules, and decoding protocols, to ensure a controlled and fair comparison of reconstruction accuracy, chemical validity, and structural fidelity. To examine the contribution of encoder design within this comparison, Table S2 in the Additional Information reports a controlled ablation of BiLSTM, GATv2, and Transformer encoders with a shared decoder, including parameter counts and teacher‐forced and beam‐search validation metrics. The corresponding differences in beam‐search chemical validity and reconstruction edit distance are visualized in Figure S2 in the Additional Information.

**TABLE 3:** Comparative Summary (latent dimension = 64).

Model	VAE [9]		TVAE [19]		Transformer‐VAE [18]		LTVAE	
Dataset	Train: 250 k, Validation: 80 k, Test: ZINC15 = 270/Dyes = 34							
Parameters^a^	∼1.92 M		∼3.72 M		∼5.49 M		∼2.78 M	
Training time	66.01 min		1224.98 min		372.18 min		353.45 min	
Test sets	ZINC15	Dye	ZINC15	Dye	ZINC15	Dye	ZINC15	Dye
Test Acc.	88.95%	73.86%	79.08%	48.98%	98.08%	92.49%	98.58%	95.56%
String Acc.	62.96%	32.35%	34.81%	5.88%	85.93%	55.88%	92.19%	61.76%
Validity ratio	83.70%	61.8%	97.81%	91.17%	97.40%	79.40%	99.30%	85.30%
Avg. Lev. Dist.	3.29	7.65	5.94	17.76	0.47	2.44	0.29	1.97

^a^ Parameters denote trainable parameters in Encoder network only.

The baseline SMILES‐based VAE, with a lightweight encoder containing 1.92 M parameters, trained rapidly (66 min) but exhibited limited reconstruction fidelity, achieving 88.95% token‐level accuracy on the ZINC15 test set and 73.86% accuracy on the dye test set. This model also showed substantial structural deviation, with average Levenshtein distances of 3.29 on ZINC15 molecules and 7.65 on dyes, indicating poor sequence‐level reconstruction, particularly under out‐of‐distribution conditions. The TVAE baseline improved reconstruction fidelity and chemical validity relative to the standard VAE; however, this improvement came at the cost of a substantially larger encoder (3.72 M parameters) and significantly longer training time (1224 min), while reconstruction accuracy on the dye test set remained limited (48.98%).

The Transformer‐VAE achieved further gains in reconstruction quality, reaching 98.08% accuracy on ZINC15 and 92.49% on dyes, with correspondingly lower structural deviations of 0.47 and 2.44, respectively, but relied on the heaviest encoder configuration (5.49 M parameters). By contrast, the proposed LTVAE achieved the most favorable trade‐off between reconstruction performance, reconstruction robustness on a structurally distinct OOD dataset, and parameter efficiency, delivering 98.58% accuracy on ZINC15 and 95.56% on the dye test set, alongside the lowest average Levenshtein distances (0.29 on ZINC15 and 1.97 on dyes) and strong chemical validity ratios (99.30% and 85.30%, respectively), all while using a significantly more compact encoder with 2.78 M parameters.

These results further indicate that latent dimensionalities of 64 and above provide an effective trade‐off between representational capacity and decoding precision. While very small latent spaces constrain reconstruction quality, increasing the dimensionality beyond 128 yields diminishing performance gains. Collectively, this analysis confirms that the proposed LTVAE architecture learns a compact yet expressive latent representation that is parameter‐efficient and maintains reconstruction performance when evaluated on chemically distinct test sets, supporting its use as a ZINC15‐trained SMILES‐to‐latent‐to‐SMILES representation model evaluated under in‐distribution and out‐of‐distribution reconstruction evaluation settings.

## 5 Conclusion

In summary, the proposed LTVAE achieved fast and stable convergence across latent dimensions, with 64–128 emerging as the most effective range, yielding high reconstruction accuracy and validity ratios near 98%–99%. Reasonable reconstruction performance was observed on held‐out ZINC15 molecules and a structurally distinct OOD dataset: on ZINC15, the model reached 98.58% reconstruction accuracy, 99.30% validity, and an average Levenshtein distance of 0.29; on dye SMILES, it achieved 95.56% accuracy, 85.30% validity, and a distance of 1.97. Property prediction from dye‐like test‐set latent vectors further demonstrated that the learned representation preserves both general molecular descriptors and dye‐relevant descriptors. In particular, the MLP achieved strong performance for ASF (MAE = 0.008, $R^{2}=0.990$) and CL (MAE = 0.487, $R^{2}=0.918$), while also predicting MW, AR, Fsp 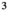, HA, and RB with high accuracy. The strong ASF and CL results directly connect the latent‐space analysis to dye‐related aromaticity and conjugation structure, and the CL‐conditioned latent traversal provides qualitative support for dye‐relevant organization of the learned manifold. Together, these results provide evidence for a ZINC15‐trained SMILES‐to‐latent‐to‐SMILES representation with reasonable reconstruction performance on both in‐distribution and OOD evaluation sets, with potential future use in downstream tasks such as screening and analysis of chemically specialized datasets, including dye libraries relevant to VOC sensing.

Despite these strengths, the current model exhibits several limitations. First, reconstruction performance degrades for very long SMILES sequences, particularly those exceeding approximately 130 characters. Such sequences often correspond to large or highly functionalized molecules, where long‐range dependency modeling and sequence truncation effects can reduce reconstruction fidelity. Second, the present evaluation focused exclusively on neutral, single‐component molecules; SMILES containing salts, counterions, or multi‐component ionic species were not explored. These representations introduce additional syntactic complexity and chemical heterogeneity that may challenge both tokenization and latent decoding. Addressing these limitations will require architectural and preprocessing extensions, such as improved handling of long‐range dependencies, explicit modeling of multi‐fragment SMILES, and ion‐normalized representations.

Future work should focus on extending the proposed framework toward chemical sensing applications, with particular emphasis on dye‐based and functional molecule libraries used in volatile organic compound (VOC) detection. In this context, incorporating salts and ionic species will be particularly important, as many sensing dyes and functional materials exist in charged or counterion‐associated forms. By leveraging the learned latent space as a chemically meaningful and compact representation, the model can be integrated into downstream pipelines for property‐guided dye design, sensor material screening, and structure–response analysis. These extensions will require additional validation and may benefit from domain‐specific fine‐tuning before broader sensing and materials discovery claims can be made.

## Author Contributions

Md Halim Mondol conceived the study, developed and implemented the proposed model, conducted the experiments and data analysis, and wrote the original manuscript. Qingshan Wei reviewed the manuscript and provided suggestions that improved the quality and presentation of the work. Edgar Lobaton supervised the research, provided methodological guidance, contributed to the study design, and reviewed and revised the manuscript. All authors reviewed and approved the final manuscript.

## Funding

This work was supported by the National Science Foundation under Grant No. 2344423.

## Conflicts of Interest

The authors declare no conflicts of interest.

## Supporting information


**Table S1:** Predictive performance of latent‐vector‐based property prediction model for all evaluated molecular descriptors.
**Figure S1:** Complete latent‐space traversal examples generated by perturbing latent vectors along learned property directions: (a) Conjugation length (CL), (b) aromatic surface fraction (ASF), and (c) molecular weight (MW). Each panel shows decoded molecules obtained from successive latent perturbations together with their corresponding measured property values, illustrating that the learned latent space encodes smooth and chemically meaningful variations in both dye‐specific and general molecular properties.
**Figure S2:** Encoder ablation results. Beam‐search reconstruction performance for different encoder architectures. (a) chemical validity ratio of reconstructed SMILES, and (b) average Levenshtein distance between reconstructed and target SMILES. All models use the same decoder, latent dimensionality, beam settings, and training protocol; only the encoder architecture is varied.
**Table S2:** Encoder ablation study.
**Figure S3:** Ablation analysis of SMILES randomization and input regularization strategies. Performance comparison of the proposed LTVAE model with and without randomized SMILES augmentation, token corruption, and label smoothing across three evaluation settings: (a) validation set, (b) ZINC15‐like test set, and (c) dye SMILES test set. Each panel reports beam‐search reconstruction accuracy, chemical validity ratio, and average Levenshtein distance. While models trained without randomization achieve slightly higher reconstruction performance on in‐distribution data, the inclusion of randomized SMILES and token‐level corruption improves generalization to the structurally distinct dye dataset, yielding higher validity and reduced structural deviation. These results indicate that encoder‐side SMILES randomization and decoder‐side input corruption enhance robustness to out‐of‐distribution molecular structures.
**Figure S4:** Assessment of out‐of‐distribution severity relative to a randomly sampled ZINC15 reference set. (a) Distribution of nearest‐neighbor Morgan fingerprint distance (1 − Tanimoto similarity) for exact, low‐error, and high‐error dye reconstructions. (b) Relationship between reconstruction error (Levenshtein distance between input and reconstructed SMILES) and nearest‐neighbor Morgan fingerprint distance. (c) Distribution of nearest‐neighbor normalized SMILES edit distance across reconstruction error groups. (d) Relationship between reconstruction error and nearest‐neighbor normalized SMILES edit distance. Neither fingerprint‐based nor sequence‐based nearest‐neighbor similarity exhibited a statistically significant association with reconstruction accuracy, indicating that reconstruction performance is not primarily determined by proximity to the sampled ZINC15 chemical space.
**Figure S5:** Correlation between reconstruction error and selected molecular descriptors. Pairwise relationships among reconstruction error (Levenshtein distance between the input and reconstructed canonical SMILES), conjugation length, aromatic surface fraction, and SMILES length for the dye dataset. Diagonal panels show the distributions of individual variables, whereas off‐diagonal panels present scatter plots with least‐squares regression lines and 95% confidence intervals. Each panel reports the Pearson correlation coefficient (*r*), Spearman rank correlation coefficient (*ρ*), and coefficient of determination (*R* ^2^). The weak correlations observed between reconstruction error and the individual molecular descriptors indicate that reconstruction performance is not dominated by any single structural property but instead reflects the combined influence of multiple molecular features.
**Table S3:** Reconstruction performance across chemically distinct PubChem molecular domains.

## Data Availability

The source code and reproduction guidelines along with datasets are available at the directed GitHub repo: https://github.com/ARoS‐NCSU/ChemInfo‐LatentRep/.
