# Supplementary material for: LTVAE: Hybrid LSTM–Transformer VAE Architecture for Molecular SMILES Representation
Source: J Comput Chem. 2026 Nov 3;47(29):e70521. doi: 10.1002/jcc.70521 (PMC13731161; doi:10.1002/jcc.70521)
Supplement: Supplementary file 1 [file JCC-47-0-s001.zip › Figure_S3.pdf]

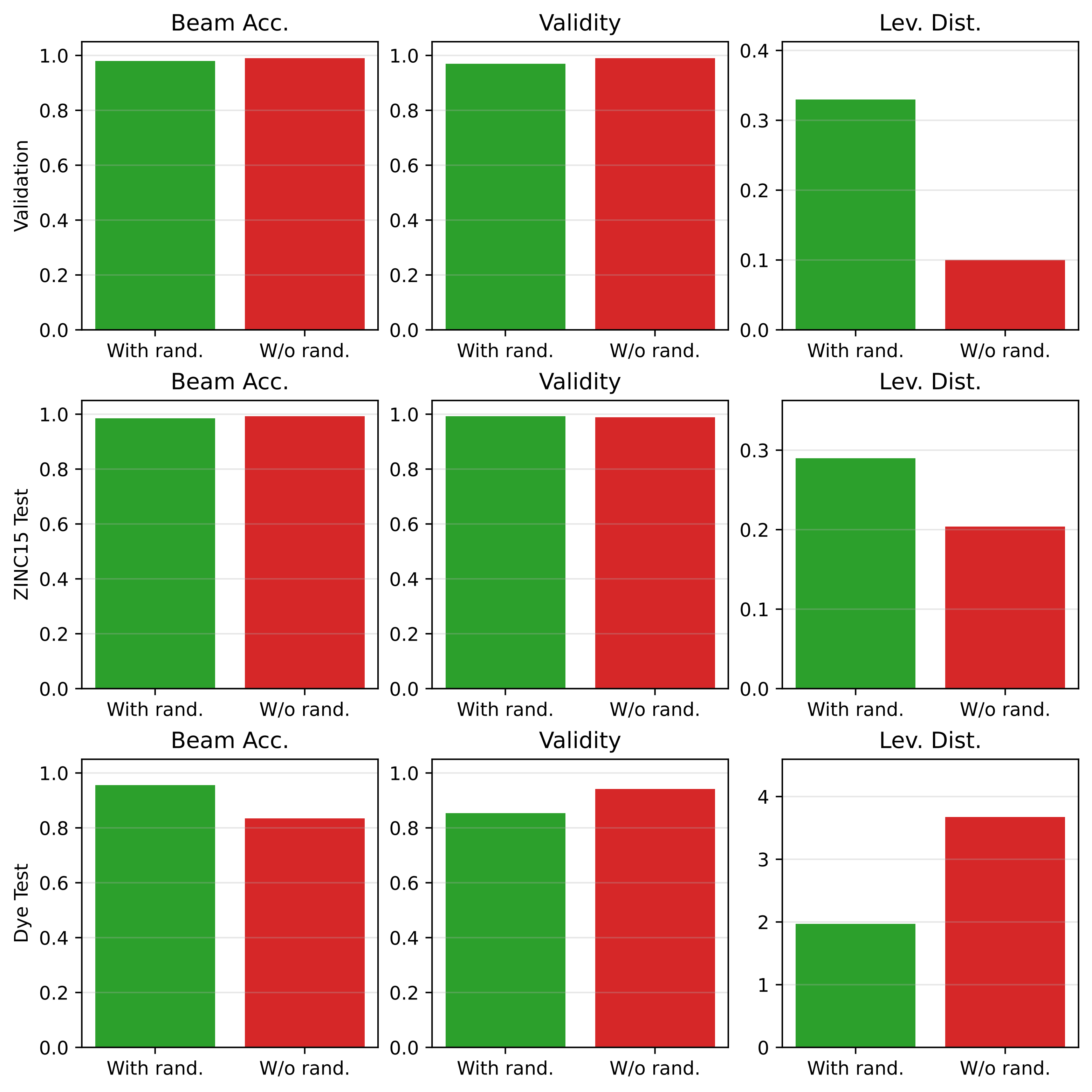
Beam Acc.
Validity
Lev. Dist.
Validation
ZINC15 Test
Dye Test
With rand.
W/o rand.
