# Supplementary material for: LTVAE: Hybrid LSTM–Transformer VAE Architecture for Molecular SMILES Representation
Source: J Comput Chem. 2026 Nov 3;47(29):e70521. doi: 10.1002/jcc.70521 (PMC13731161; doi:10.1002/jcc.70521)
Supplement: Supplementary file 1 [file JCC-47-0-s001.zip › jcc70521-sup-0001-Supinfo.docx]

Additional Information

**PROPERTY PREDICTION OUTCOMES**

To provide a comprehensive evaluation of the latent representations learned by the proposed LTVAE model, Table S1 summarizes the predictive performance for all investigated molecular properties using latent vectors as input to downstream property prediction models. Both general molecular descriptors (e.g., molecular weight, aromatic rings, heavy atom count, Fsp³, hydrogen-bond acceptors, hydrogen-bond donors, topological polar surface area, rotatable bonds, logP, and QED) and dye-relevant descriptors (conjugation length and aromatic surface fraction) are included. Model performance is reported using the coefficient of determination (R²) and mean absolute error (MAE), allowing direct comparison of how accurately different physicochemical properties are encoded within the learned latent space.

**Table S1.** Predictive performance of latent-vector-based property prediction model for all evaluated molecular descriptors.

| **Properties** | **MAE_ridge** | **R^2^_ridge** | **MAE_mlp** | **R^2^_mlp** |
| --- | --- | --- | --- | --- |
| AR | 0.153566 | 0.817279 | 0.004804 | 0.997571 |
| ASF | 0.024010 | 0.919769 | 0.007827 | 0.990261 |
| Fsp^3^ | 0.032878 | 0.897494 | 0.014048 | 0.979987 |
| HA | 0.478550 | 0.880910 | 0.201429 | 0.977611 |
| RB | 0.717283 | 0.622800 | 0.303792 | 0.924248 |
| CL | 1.411916 | 0.420390 | 0.486715 | 0.917908 |
| MW | 12.585444 | 0.479811 | 5.229163 | 0.899432 |
| HBD | 0.284596 | 0.187028 | 0.085956 | 0.849328 |
| QED | 0.045674 | 0.477043 | 0.024310 | 0.836272 |
| HBA | 0.501873 | 0.317544 | 0.245148 | 0.820959 |
| TPSA | 6.404595 | 0.286692 | 3.496332 | 0.773604 |
| LogP | 0.341404 | 0.139634 | 0.211691 | 0.662299 |

**STUDY ON LATENT-SPACE TRAVERSAL**

(a)

(b)

(c)

**Figure S1.** Complete latent-space traversal examples generated by perturbing latent vectors along learned property directions: (a) Conjugation length (CL), (b) aromatic surface fraction (ASF), and (c) molecular weight (MW). Each panel shows decoded molecules obtained from successive latent perturbations together with their corresponding measured property values, illustrating that the learned latent space encodes smooth and chemically meaningful variations in both dye-specific and general molecular properties.

To further illustrate the organization of the learned latent space, Figure S1 presents complete latent-space traversal experiments performed along three representative property directions. Traversals were conducted by moving a latent vector along directions learned for conjugation length (CL), aromatic surface fraction (ASF), and molecular weight (MW), followed by decoding each modified latent vector into molecular structures. The measured property values of the decoded molecules demonstrate gradual and interpretable changes, providing qualitative evidence that the latent space captures chemically meaningful directions corresponding to both general and dye-relevant molecular characteristics. Such behavior supports the suitability of the proposed latent representation for property-aware molecular manipulation and downstream applications requiring interpretable structure–property relationships.

**ABLATION STUDY**

1. **Encoder Ablation**

To assess the influence of the encoder architecture on model performance, an encoder ablation study was conducted in which only the encoder component of the variational autoencoder was varied, while all other aspects of the model were held constant. Three encoder types were considered: a bidirectional LSTM operating on tokenized SMILES strings (LTVAE), a graph-based encoder employing GATv2 layers applied to molecular graphs (TVAE) [1], and a Transformer encoder applied to SMILES sequences (Transformer-VAE) [2]. In all cases, the decoder architecture, latent dimensionality, training dataset, optimization schedule, and decoding strategy were identical.

|   (a) |  (b) |
| --- | --- |
| **Figure S2.** Encoder ablation results. Beam-search reconstruction performance for different encoder architectures. (a) chemical validity ratio of reconstructed SMILES, and (b) average Levenshtein distance between reconstructed and target SMILES. All models use the same decoder, latent dimensionality, beam settings, and training protocol; only the encoder architecture is varied. | |

**Table S2:** Encoder ablation study.

| **Model** | **Encoder Type** | **Parameters** | **Val Acc. (TF)** | **Val Acc. (Beam)** | **Validity (Beam)** | **Avg. Lev. Dist. (Beam)** |
| --- | --- | --- | --- | --- | --- | --- |
| TVAE [1] | GATv2 | 3.72M | 96.60% | 78.90% | 98.10% | 5.60 |
| Transformer-VAE [2] | Transformer | 5.54M | 99.20% | 96.90% | 95.70% | 0.66 |
| LTVAE | BiLSTM | 2.78M | 99.60% | 98.10% | 97.80% | 0.33 |

The results, summarized in Table S2 and Figure S2, indicate that the choice of encoder has a measurable impact on reconstruction quality. The graph-based encoder yields a higher fraction of chemically valid reconstructions and a lower average Levenshtein distance relative to the sequence-based encoders. This behavior suggests that incorporating explicit molecular graph information in the encoder facilitates the learning of latent representations that better preserve chemically relevant structural features. The Transformer encoder exhibits intermediate performance between the BiLSTM and graph-based encoders, indicating that increased sequence modeling capacity alone does not fully compensate for the absence of explicit structural information.

2. **Bottleneck Ablation: Effect of Latent Dimensionality**

The effect of the variational bottleneck on reconstruction fidelity and latent expressiveness was examined by systematically varying the latent dimensionality of the proposed LTVAE architecture. This analysis is summarized in Figure 3 of the main manuscript, where reconstruction accuracy, chemical validity ratio, and average Levenshtein distance are reported across training epochs for latent dimensions of 4, 8, 16, 32, 64, 128, and 256. All experiments were conducted using the same encoder–decoder architecture, dataset, and training protocol, ensuring that observed performance differences arise solely from changes in bottleneck capacity.

As shown in Figure 3, very small latent spaces (latent dimension ≤ 16) exhibit limited representational capacity, resulting in substantially lower reconstruction accuracy and persistently higher structural deviation. A pronounced transition is observed between latent dimensions of 16 and 32, where reconstruction accuracy improves sharply and Levenshtein distance decreases rapidly. This behavior indicates that this range represents the minimum latent capacity required to reliably encode chemically meaningful structure. For larger latent dimensionalities (≥ 64), reconstruction accuracy and validity approach saturation, while further increases in latent size provide only marginal improvement, reflecting diminishing returns in representational scaling.

Overall, this bottleneck ablation demonstrates that the proposed LTVAE framework preserves sufficient molecular information even with compact latent representations, and that a moderate latent dimensionality (e.g., 64) provides an effective balance between reconstruction fidelity, latent compactness, and computational efficiency. These results directly support the architectural design choices adopted in the main manuscript.

3. **Ablation on Randomized SMILES & Token Corruption**

To assess the impact of SMILES randomization and input-level regularization on model robustness, an ablation study was performed comparing two training configurations of the proposed LTVAE architecture. In the first configuration, encoder-side randomized SMILES augmentation was combined with decoder-side token corruption and label smoothing. In the second configuration, all three mechanisms were disabled, and the model was trained exclusively on canonical SMILES without input noise.

As shown in Figure S3 models trained without randomization exhibit faster convergence and marginally improved reconstruction performance on in-distribution ZINC15-like data, achieving slightly higher token-level accuracy and lower average Levenshtein distance. This behavior reflects tighter memorization of canonical SMILES representations when no stochastic augmentation is applied.

In contrast, evaluation on the dye SMILES test set reveals a consistent advantage for the randomized training configuration. Models trained with SMILES randomization and token corruption achieve higher reconstruction accuracy, improved chemical validity, and reduced structural deviation relative to their non-randomized counterparts. These gains indicate enhanced robustness to SMILES ordering and improved generalization to chemically distinct molecules that are sparsely represented in large-scale drug-like datasets.

Overall, the ablation results highlight a trade-off between optimal in-distribution reconstruction and out-of-distribution generalization. While noise-free training favors ZINC-like molecules, the inclusion of randomized SMILES and token-level corruption produces a more stable and transferable latent representation. Given the target application to specialized dye chemistries, the randomized training strategy was retained in the final model configuration.

**Figure S3.** Ablation analysis of SMILES randomization and input regularization strategies. Performance comparison of the proposed LTVAE model with and without randomized SMILES augmentation, token corruption, and label smoothing across three evaluation settings: (a) validation set, (b) ZINC15-like test set, and **(c)** dye SMILES test set. Each panel reports beam-search reconstruction accuracy, chemical validity ratio, and average Levenshtein distance. While models trained without randomization achieve slightly higher reconstruction performance on in-distribution data, the inclusion of randomized SMILES and token-level corruption improves generalization to the structurally distinct dye dataset, yielding higher validity and reduced structural deviation. These results indicate that encoder-side SMILES randomization and decoder-side input corruption enhance robustness to out-of-distribution molecular structures.

**OUT-OF-DISTRIBUTION SEVERITY ANALYSIS**

To further examine whether reconstruction accuracy was primarily influenced by molecular novelty, each dye molecule was compared with a random reference sample of 100,000 molecules from the ZINC15 database [3]. Two complementary similarity measures were evaluated. First, Morgan fingerprint Tanimoto similarity was used to identify the nearest structural neighbor within the reference sample. Second, the normalized Levenshtein distance between canonical SMILES strings was calculated to quantify sequence-level similarity to the nearest reference molecule. Because the exact 250,000-molecule training subset was not retained after model development, these analyses were performed relative to a randomly sampled ZINC15 reference set rather than the original training molecules.

As shown in Figure S4, neither Morgan fingerprint similarity nor normalized SMILES edit distance exhibited a statistically significant association with reconstruction accuracy (two-sided Mann–Whitney U test, *p* = 0.172 and *p* = 0.357, respectively, where *p* denotes the probability of observing an equal or more extreme difference under the null hypothesis). Likewise, correlation analysis revealed only weak relationships between reconstruction error and the corresponding OOD measures (Morgan distance: Pearson correlation coefficient, *r* = -0.10, and Spearman rank correlation coefficient, *ρ* = -0.21; normalized SMILES edit distance: Pearson *r* = -0.13 and Spearman *ρ* = -0.16).

The distributions of both fingerprint- and sequence-based similarity measures are presented in Figure S4. Exact, low-error, and high-error reconstruction groups exhibit substantial overlap in both nearest-neighbor fingerprint similarity and normalized SMILES edit distance, with no consistent increase in reconstruction error as molecular distance from the sampled ZINC15 reference set increases. Collectively, these results indicate that reconstruction accuracy is not primarily governed by global proximity to the sampled ZINC15 chemical space. Instead, when considered alongside the analyses presented in the main manuscript, they support the interpretation that reconstruction difficulty is more closely associated with intrinsic structural characteristics, particularly conjugation length and molecular flexibility, than with overall out-of-distribution distance.

(a)

(b)

(c)

(d)

**Figure S4.** Assessment of out-of-distribution severity relative to a randomly sampled ZINC15 reference set. (a) Distribution of nearest-neighbor Morgan fingerprint distance (1 − Tanimoto similarity) for exact, low-error, and high-error dye reconstructions. (b) Relationship between reconstruction error (Levenshtein distance between input and reconstructed SMILES) and nearest-neighbor Morgan fingerprint distance. (c) Distribution of nearest-neighbor normalized SMILES edit distance across reconstruction error groups. (d) Relationship between reconstruction error and nearest-neighbor normalized SMILES edit distance. Neither fingerprint-based nor sequence-based nearest-neighbor similarity exhibited a statistically significant association with reconstruction accuracy, indicating that reconstruction performance is not primarily determined by proximity to the sampled ZINC15 chemical space.

**DESCRIPTOR-LEVEL ANALYSIS OF RECONSTRUCTION ERROR**

To further investigate which molecular characteristics are associated with reconstruction difficulty, pairwise relationships between reconstruction error (Levenshtein distance between the input and reconstructed canonical SMILES) and selected molecular descriptors were examined (Figure S5). The analysis includes conjugation length, aromatic surface fraction, and SMILES length, together with their mutual correlations. Pearson correlation coefficients (r), Spearman rank correlation coefficients (ρ), and coefficients of determination (R²) are reported for each pairwise comparison.

**Figure S5.** Correlation between reconstruction error and selected molecular descriptors. Pairwise relationships among reconstruction error (Levenshtein distance between the input and reconstructed canonical SMILES), conjugation length, aromatic surface fraction, and SMILES length for the dye dataset. Diagonal panels show the distributions of individual variables, whereas off-diagonal panels present scatter plots with least-squares regression lines and 95% confidence intervals. Each panel reports the Pearson correlation coefficient (r), Spearman rank correlation coefficient (ρ), and coefficient of determination (R²). The weak correlations observed between reconstruction error and the individual molecular descriptors indicate that reconstruction performance is not dominated by any single structural property but instead reflects the combined influence of multiple molecular features.

As shown in Figure S5, none of the examined descriptors exhibits a strong linear relationship with reconstruction error. Conjugation length shows a weak positive association with reconstruction error (r = 0.24, ρ = 0.40), whereas aromatic surface fraction (r = −0.28) and SMILES length (r = −0.34) display weak negative trends. The corresponding coefficients of determination remain low (R² ≤ 0.12), indicating that each descriptor individually explains only a small fraction of the observed reconstruction variability. These results suggest that reconstruction accuracy is not governed by any single molecular property. Instead, reconstruction difficulty is likely influenced by the combined effects of multiple structural characteristics, consistent with the observations reported in the main manuscript.

**PERFORMANCE ACROSS CHEMICALLY DISTINCT PUBCHEM MOLECULAR DOMAINS**

To further examine the generalization capability of the proposed model, reconstruction performance was evaluated using approximately 20,000 molecules collected from five chemically distinct PubChem molecular domains [4], including drug-like compounds, pigment indicators, natural products, polymer monomers, and protein–enzyme chemistry molecules. These datasets were not used during model training and were processed using the same preprocessing pipeline adopted throughout this work. The reconstruction results for each molecular domain are summarized in Table S3.

**Table S3:** Reconstruction performance across chemically distinct PubChem molecular domains.

| **Test Sets** | **Test Acc.** | **String Acc.** | **Validity** | **Avg. Lev. Dist.** |
| --- | --- | --- | --- | --- |
| Drug-like | 82.10% | 41.53% | 77.35% | 4.36 |
| Pigment indicator | 65.89% | 3.94% | 50.06% | 9.45 |
| Natural products | 88.37% | 61.91% | 84.90% | 2.38 |
| Polymers monomers | 79.98% | 46.60% | 84.54% | 2.35 |
| Protein-enzyme chemistry | 81.58% | 49.80% | 83.25% | 4.15 |
| Overall | 79.57 ± 8.28% | 40.76 ± 21.91% | 76.02 ± 14.83% | 4.54 ± 2.91 |

As expected, reconstruction performance varied across the five molecular domains, reflecting differences in their structural characteristics and chemical composition. Natural products exhibited the highest reconstruction accuracy, whereas pigment indicator molecules were the most challenging. Such variation is expected because the model was trained exclusively on ZINC15 molecules and was not optimized for any of these application-specific chemical domains.

Overall, the model achieved a mean token-level reconstruction accuracy of 79.57 ± 8.28%, a string-level reconstruction accuracy of 40.76 ± 21.91%, a validity of 76.02 ± 14.83%, and an average Levenshtein distance of 4.54 ± 2.91 across the five PubChem molecular domains. These results suggest that the proposed LTVAE learns general chemical representations that remain applicable across multiple chemically distinct molecular domains, even without domain-specific training. They also indicate that, for specialized application areas, domain-specific fine-tuning would likely further improve reconstruction accuracy and better adapt the learned representation to the target chemical space.

**REFERENCES**

[1] Yoshikai, Y.; Mizuno, T.; Nemoto, S.; Kusuhara, H. A novel molecule generative model of VAE combined with Transformer. arXiv preprint arXiv:2402.11950 2024.

[2] Bhadwal, A. S.; Kumari, M.; Choudhary, S. TVAE: Transformer Based Variational Autoencoder for the Design of Drug Molecules. Proceedings of the International Conference on Computer, Electronics, Electrical, Engineering & their Applications (IC2E3). 2025.

[3] John J Irwin, Teague Sterling, Michael M Mysinger, Erin S Bolstad, and Ryan G Coleman. Zinc: a free tool to discover chemistry for biology. Journal of chemical information and modeling, 52(7):1757–1768, 2012.

[4] Kim, S., Chen, J., Cheng, T., Gindulyte, A., He, J., He, S., Li, Q., Shoemaker, B. A., Thiessen, P. A., Yu, B., Zaslavsky, L., Zhang, J., & Bolton, E. E. (2023). PubChem 2023 update. *Nucleic Acids Research, 51*(D1), D1373–D1380.
